# Improving the Thermal and Mechanical Properties of Poly(l-lactide) by Forming Nanocomposites with an in Situ Ring-Opening Intermediate of Poly(l-lactide) and Polyhedral Oligomeric Silsesquioxane

**DOI:** 10.3390/nano9050748

**Published:** 2019-05-15

**Authors:** Xiu-Xiu Lei, Hao Lu, Lei Lu, Hai-Qing Xu, Ying-Guo Zhou, Jun Zou

**Affiliations:** 1 School of Materials Science and Engineering, Jiangsu University of Science and Technology, Zhenjiang 212003, China; m18261956361@163.com (X.-X.L.); luhao1885@163.com (H.L.); lulei19941027@163.com (L.L.); 2 Jiangsu Provincial Engineering Laboratory for Advanced Materials of Salt Chemical Industry, Huaiyin Institute of Technology, Huai’an 223003, China; xuhaiqing@hyit.edu.cn

**Keywords:** poly(l-lactide) (PLLA), ring-opening polymerization, compatibility, nanocomposites, (3-amino)-propylheptaisobutyl cage silsesquioxane (AMPOSS)

## Abstract

In this study, a series of poly(l-lactide) and (3-amino)-propylheptaisobutyl cage silsesquioxane (PLLA-AMPOSS) intermediates were first fabricated using single-arm in situ solution polymerization of LLA monomers and AMPOSS nanoparticles with different contents, 0.02–1.00 mol%. Then, the PLLA-AMPOSS intermediate with 0.5 mol% AMPOSS was selected as a representative and investigated by nuclear magnetic resonance (NMR) and X-ray diffraction (XRD). Afterwards, it was added into the pure PLLA with different mass fractions. Finally, the thermal behavior, crystallization kinetics, morphological characteristics, and mechanical properties of the obtained PLLA/PLLA-AMPOSS nanocomposites were carefully measured and investigated by differential scanning calorimetry (DSC), polarizing microscopy (POM), scanning electron microscopy (SEM), and tensile test. After comparing the PLLA-AMPOSS intermediate and PLLA/AMPOSS blend, the results show that the ring-open polymerization of PLLA-AMPOSS intermediate was successful. The results also show that the existence of PLLA-AMPOSS has a strong influence on the crystallization behavior of PLLA/PLLA-AMPOSS composites, which can be attributed to the heterogeneous nucleation effect of PLLA-AMPOSS. In addition, it was also found from the tensile test results that the addition of the PLLA-AMPOSS nanofiller improved the tensile strength and strain at break of PLLA/PLLA-AMPOSS nanocomposites. All of these results indicate the good nucleating effect of PLLA-AMPOSS and that the AMPOSS disperses well in the PLLA/PLLA-AMPOSS nanocomposites. A conclusion can be drawn that the selective nucleating agent and the combined method of in situ ring-opening polymerization and physical blending are feasible and effective.

## 1. Introduction

It has been well realized that environmental pollution has become significantly serious nowadays, and it is partially caused by the abandoning of traditional nondegradable materials. Degradable and recyclable polymers show increasing application potential and have become candidates to replace petroleum-based polymers. Poly(l-lactide) (PLLA), a typical biocompatible, biodegradable, renewable, and nontoxic thermoplastic polymer, has attracted much attention for investigation [1,2,3,4,5,6]. PLLA has been widely used in various fields such as packaging, medical devices, and biological engineering applications for its excellent thermal plasticity and easy processability [7,8,9]. However, its poor thermal resistance and mechanical properties have somewhat limited its wider application [10,11]. PLLA is a typical semicrystalline material, and its properties depend largely on its crystallinity and crystal structure [12]. Therefore, similar to other semicrystalline polymers, including isotactic polypropylene (iPP) [13,14,15,16], polycaprolactone (PCL) [17], poly(vinylidene fluoride) (PVDF) [18], and poly(cyclohexylene dimethylene cyclohexanedicarboxylate) (PCCE) [19,20], controlling crystallization has gradually become known as an effective way to obtain the required performance of PLLA parts. These works can be roughly classified into three categories when considering large-scale industrial applications. The first is to incorporate a plasticizer agent into the PLLA matrix and form blends, such as poly(ethylene glycol) (PEG) [21,22], glycerol [23], poly(caprolactone) (PCL) [24], poly(epsilon-caprolactone-co-l-lactide) (sPCLA) [25], oligo(d-lactic acid)-grafted cellulose [26], and poly(propylene glycol) (PPG) [27]. However, the existence of these plasticizers cannot actually improve the crystallization rate of pure PLLA, and it is even the opposite in some cases. Another approach is to adjust the processing conditions [28,29], which has been reported to have an obvious effect on the crystallization process and the ultimate crystallinity. It is well known that the amorphous phase will appear if the quench temperature is very low and the crystals have no time to form. However, improving the crystallinity is often not effective if only the temperature gradient and cooling rate are controlled, and the temperature cannot be controlled easily in real manufacturing. The final and maybe the most promising approach is to add some organic and/or inorganic particles as nucleating agents to the PLLA matrix, such as sodium stearate [30], nano-clays [31], graphite particles [32], triclosan nanoparticles [33], tungsten disulphide inorganic nanotubes (INT-WS_2_) [34], or magnesium oxide nanoparticles [35]. Compounding with these particles by physical blending is a conventional method of fabricating PLLA composites and was proved to be convenient to improve the crystallization behavior of PLLA. However, the agglomeration of nanoparticles easily affects the efficiency and effects. How to improve the dispersion and compatibility of nanoparticles in the PLLA matrix during nanoparticle filling is a general concern.

**Figure 1:**
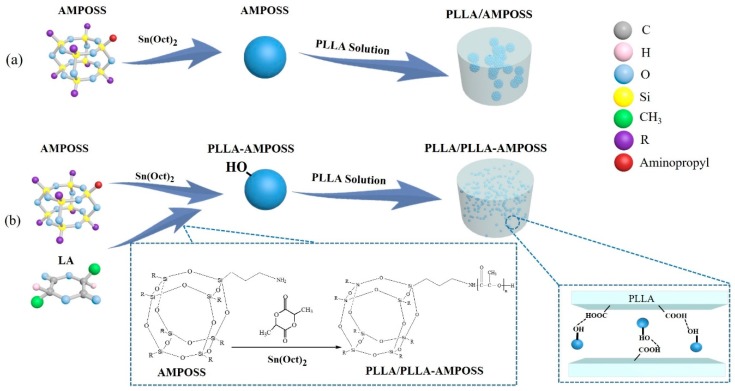
Comparison of fabrication method of poly(l-lactide) and (3-amino)-propylheptaisobutyl cage silsesquioxane (PLLA/AMPOSS) blend and PLLA/PLLA-AMPOSS nanocomposites: (**a**) conventional method using solution blending; (**b**) combined method of single-arm ring-opening polymerization and solution blending.

Polyhedral oligomeric silsesquioxane (POSS), one member of the silsesquioxane family, is a type of intermediate cubic polyhedron that has a molecular weight up to 1000 and a nanoscale cage structure. The nanosized cage structure of POSS comprises both inorganic and organic substituents that form a framework of silicon and oxygen atoms and branched chains composed by a hydrocarbon group or a polar functional group [36]. By adjusting the substituents of the POSS cage block at the molecule level in the processes of copolymerization, polycondensation, homopolymerization, and physical blending, the intermediate and composited materials with required functionalization can be fabricated and manufactured in a controlled way. In addition, an important feature of POSS is that its Si-O-Si bond, which has a diameter of 1.5 nm, can build an inorganic framework with the reactive and nonreactive groups when it is added to an appropriate polymer, which can easily control the compatibility between POSS and the polymer matrices. Hence, the POSS can be inserted into the polymer and function as a nanofiller in processes such as copolymerization, grafting reaction, and in situ polymerization, resulting in substantial improvements in thermal stability, crystallinity, and barrier properties of the polymeric materials [37,38,39]. However, the bond–bond force in the polymer matrix generally stays at the molecular scale and is not effective enough, so the interface adhesion between the POSS and the polymer matrix still needs to be improved. Furthermore, the nanosized POSS particles are difficult to disperse uniformly into the polymer matrix in the melt and solution blending processes. As shown in Figure 1a, it is easily observed that the POSS nanoparticles agglomerate after the solution blending. 

In order to improve the compatibility between the POSS particles and the polymer matrices, many attempts have been made using a suitable process of polymerization other than simple physical blending. The POSS particle was first used as a precursor in the synthesis of some kinds of nanohybrid materials such as amines [40], alkyls [41], aminos, and bromophenyls [42,43]. It is known that oxytrol and amino groups can accelerate the ring-opening polymerization of lactide monomers when they are used as initiators [44]. Therefore, the POSS nanoparticles, which have a core-shell type containing amino groups, were directly dispersed in PLA-based systems using in situ polymerization, which was found to be feasible and relied on the functionalized groups connected with the octa-POSS [45]. The developing hydrogen bond of the hydroxy group of PLLA resin can react with the amino group of POSS particles, and the graft structure between the POSS and the matrix is an interface interaction, leading to a more stable microstructure of the composites and extreme boosting of the glass transition temperature and improving melt flow behavior. As a result, it was reported that the elongation at break of PLLA/POSS composites increased approximately 10-fold compared to that of pure PLLA resin when other physical properties remained unchanged [46]. Apart from the PLA matrix, POSS nanoparticles can also be introduced into other polyester polymers, such as polybutylene succinate (PBS) and poly(butyleneadipate-co-terephthalate) (PBAT), by triggering open-ring polymerization to influence the various properties of the composites. However, it must be noted that a negative correlation between the content of the POSS particles and the number of molecules of the polymerized materials was also found [47]. This is attributed to the macromolecule chains of the polymerized materials being deeply influenced by the number of crosslinking points provided by the end group of silsesquioxane. This means that the content of the POSS particles in the composite cannot easily control the number of molecules of polymerized materials if the nanosized POSS particles are not dispersed uniformly. 

Combining ring-opening polymerization and physical blending can be viewed as a revolutionary method to further improve the interfacial interactions between POSS nanoparticles and PLLA matrices. The representative work was performed by Liu and Lu et al. [48]. They synthesized eight branched PLLA arms and a three-dimensional POSS structure with a cube-like core via ring-opening polymerization initiated by octa(3-hydroxypropyl)-POSS, and then compounded the obtained POSS-(PLLA)8 with the PLLA matrix. The obtained PLLA/POSS-(PLLA)8 composite was found to have dramatically increased mechanical properties compared with the neat PLLA resin. However, the requirement of anionic polymerization is relatively strict during the multi-arm ring-opening polymerization of the eight-branched POSS-PLLA composites. In addition, the eight arms were somewhat short, so the chain mobility of the obtained POSS-PLLA composites was probably limited, and hence the crystallization of the PLLA was influenced and blocked. 

In this study, we propose a novel method to fabricate PLLA and POSS nanocomposites by combining in situ ring-opening polymerization and solution blending. For the first time, we applied nanoparticles of (3-amino) propylheptaisobutyl cage silsesquioxane POSS (AMPOSS) as an initiator and stannous (II) octoate (Sn(Oct)_2_) as a catalyst to trigger the ring-opening polymerization of l-lactide monomers. A large number of intermediates with different mass fractions were prepared and manufactured with the aim of producing a reactive functional group. The obtained PLLA-POSS was then incorporated into the pure PLLA homopolymers to form PLLA/PLLA-AMPOSS composites by means of solution blending. Moreover, to explore the reactive mechanism and modified effect, the microscopic chemical structures of the PLLA-POSS intermediate and PLLA/PLLA-AMPOSS composite were characterized by ^1^H-NMR spectroscopy and X-ray diffraction (XRD) analysis. The thermal properties, crystallization kinetics, crystallization morphology, and distribution of the dispersed phase of the PLLA/PLLA-AMPOSS composite were also investigated by thermogravimetric analysis (TGA), differential scanning calorimetry (DSC), and scanning electron microscopy (SEM), respectively. In addition, the mechanical properties of the obtained nanocomposite were tested and compared by the tensile test. 

## 2. Experimental

### 2.1. Materials

Poly (l-lactide) (PLLA, M_n_ = 1.0 × 10^5^g/mol) used in this study was a commercial-grade product purchased from Jiuding Biological Engineering Co., Ltd. (Nantong, China), and l-lactide was self-made in our laboratory with a residual monomer content less than 0.5 wt%. Polyhedral oligomeric silsesquioxane (POSS) nanoparticles were obtained from Hybrid Plastics Co., Ltd., Hattiesburg, MI, USA. Stannous (II) octoate (Sn(Oct)_2_, 95%) was obtained from Macklin Biochemical Co., Ltd. (Shanghai, China). Dichloromethane (CH_2_CL_2_), dried methyl alcohol, and ethyl formate (CH_3_COOC_2_H_5_) were kindly supplied by Sinopharm Chemical Reagent Co., Ltd., Shanghai, China, and were of analytical grade, and there was no need for further purification. 

### 2.2. Sample Preparation

The sample preparation was realized in two main stages, synthesis of PLLA-AMPOSS intermediates by single-arm ring-opening polymerization and fabrication of PLLA/PLLA-AMPOSS nanocomposites. 

#### 2.2.1. Synthesis of PLLA-AMPOSS Intermediates by Ring-Opening Polymerization

The fabrication of PLLA-AMPOSS intermediates was separated into the following steps. First, the l-lactide (LLA) monomer was purified by recrystallization 3 times in a solution of ethyl acetate. Then, the purified LLA monomer was added to a flask ampoule that contained xylene solution. The ampoule was flame-dried and equipped with a stirring bar before use. Following that, the AMPOSS nanoparticles were added to the LLA solution, and its mole ratio to LLA was fixed at 0.02%, 0.05%, 0.10%, 0.20%, 0.50%, and 1.00%. Subsequently, a Sn(Oct)_2_ solution was injected into the ampoule, and its usage was 1/20,000 of the LLA monomer in mole fraction. After that, the reaction vessel was transferred to an oil bath at 120 °C under vigorous stirring until the mixture thoroughly melted. Then, the mixture was heated to 150 °C with the help of magnetic stirring under an atmosphere of dry nitrogen, and the LLA monomer and AMPOSS nanoparticles began to undergo ring-opening polymerization. After the reaction lasted for 6 h, the products were rapidly quenched using a refrigerator to terminate polymerization. The final reaction products, which were named PLLA-AMPOSS_x_ intermediate, were dissolved in chloroform, precipitated into excess methanol, and filtrated and dried in vacuum at 50 °C for 24 h. The subscript of PLLA-AMPOSS_x_ intermediate denotes the molar percentage of AMPOSS to LLA monomer. A series of intermediates were thus obtained: PLLA-AMPOSS_0.02_, PLLA-AMPOSS_0.05_, PLLA-AMPOSS_0.1_, PLLA-AMPOSS_0.2_, PLLA-AMPOSS_0.5_, and PLLA-AMPOSS_1.0_. The reaction scheme used in this study is shown in Figure 1b. 

#### 2.2.2. Fabrication of PLLA/PLLA-AMPOSS Nanocomposites

The obtained PLLA-AMPOSS_0.50_ intermediate was then used to fabricate PLLA/PLLA-AMPOSS nanocomposites. An optional synthesis procedure of the PLLA/PLLA-AMPOSS nanocomposites can be described as follows. Pure PLLA resin and PLLA-AMPOSS_0.50_ intermediate were dissolved in dichloromethane solution sequentially with vigorous magnetic stirring, and the content of PLLA-AMPOSS_0.50_ intermediate was 1%, 5%, 10%, 20%, and 30% in weight. Subsequently, the solution was precipitated with overdosed methanol, and the obtained nanocomposites were dried in vacuo at 50 °C for 24 h. The nanocomposites were named PLLA/PLLA-AMPOSS^y^, where the superscript y represents the weight percentage of PLLA-AMPOSS_0.50_ intermediate. There were 5 kinds of PLLA/PLLA-AMPOSS nanocomposites used in this study: PLLA/PLLA-AMPOSS^1^, PLLA/PLLA-AMPOSS^5^, PLLA/PLLA-AMPOSS^10^, PLLA/PLLA-AMPOSS^20^, and PLLA/PLLA-AMPOSS^30^. For conventional comparison, simple solution blends of PLLA resin and AMPOSS nanoparticles with content of 0.1 wt% and 1.0 wt%, respectively, were also prepared using the traditional method, as shown in Figure 1a, and named as PLLA/AMPOSS blends: PLLA/0.1wt%AMPOSS and PLLA/1.0wt%AMPOSS depending on the content of AMPOSS. The PLLA/1.0wt%AMPOSS and PLLA/0.1wt%AMPOSS blends were used to compare with the PLLA-AMPOSS intermediates and PLLA/PLLA-AMPOSS nanocomposites, respectively. 

### 2.3. Sample Tests

Proton nuclear magnetic resonance (^1^H-NMR) spectra were probed by a 600 MHz NMR spectrometer (Bruker AVANCE II 600 MHz, Madison, WI, USA) in deuterated chloroform (CDCl_3_) at room temperature, with tetramethylsilane (TMS) used as the internal reference. 

X-ray diffraction patterns were obtained with an XRD-6000 diffractometer (Shimadzu Co., Kyoto, Japan) equipped with Ni-filtered Cu Kα radiation (wavelength, λ = 0.154 nm), running at 40 kV and 200 mA at a scanning rate of 4.0° min^−1^ and with a scanning range 2θ = 5–40°. All the samples were heated to 200 °C, held for 5 min, and cooled down to 110 °C at a cooling rate of 100 °C/min until the crystallization of PLLA was completed. 

The thermal properties of the sample, approximately 5 mg of the fabricated PLLA/PLLA-AMPOSS nanocomposites, was recorded by differential scanning calorimeter (DSC 204 F1 Phoenix, NETZSCH, Bavaria, Germany) under a nitrogen purge. The sample was heated to 220 °C at a rate of 10 °C/min and held for 5 min to eliminate the thermal history, and then cooled down to room temperature at a rate of 40 °C/min. The sample was heated from room temperature to 220 °C at a rate of 10 °C/min for the second time. In addition, to investigate the isothermal crystallization kinetics, after being heated to 220 °C at a rate of 20 °C/min and held for 5 min, the sample was cooled down to the desired crystallization temperature (*T*_c_) at a rate of 40 °C min^−1^ until the isothermal crystallization process was completed. All exothermal traces were collected and analyzed. 

A thermal gravimetric analyzer (TGA) (Pyris Diamond, Perkin Elmer, Waltham, MA, USA) was used to investigate the thermal stability of the sample under nitrogen and air atmosphere. The sample, with a mass of 20 mg, was heated from room temperature to 600 °C at a rate of 10 °C/min. 

Spherulitic morphology image of the PLLA/PLLA-AMPOSS nanocomposite was observed directly using polarized optical microscopy (POM) (50iPOL, Nikon, Tokyo, Japan) with a hot stage. The selective samples were initially sandwiched in 2 cover glasses, then melted at 200 °C and compressed in a film for 3 min to remove the thermal-mechanical history. Following that, the samples were cooled down to 125 °C with a high cooling rate, and kept for the necessary time until the crystallization was completed. A comparison of the crystal’s morphology over 40 s and 200 s was recorded. 

A scanning electron microscope (SEM, XL-30; Philips, Amsterdam, Holland) was used to examine the compatibility and dispersion status of the AMPOSS nanoparticles. The PLLA-AMPOSS blend and PLLA/PLLA-AMPOSS nanocomposites were immersed in liquid nitrogen for about 1 h and then freeze-fractured. The fractured surfaces were sputtered with gold before observation. 

Tensile tests were carried out according to ASTM D638-10 standards, using a screw-driven universal testing instrument (CMT-4303, Chengde, China) under room conditions. A crosshead speed of 50 mm/min was used to study the stress and strain behavior of the molded samples. Seven tensile bars were tested for each formula, and the biggest and smallest values were excluded. Hence, 5 values were selected for analysis, and the mean and range of ultimate tensile strength and strain at break for each group of samples were calculated and reported. 

## 3. Results and Discussion

### 3.1. Characterization of PLLA-AMPOSS Intermediates

**Figure 2:**
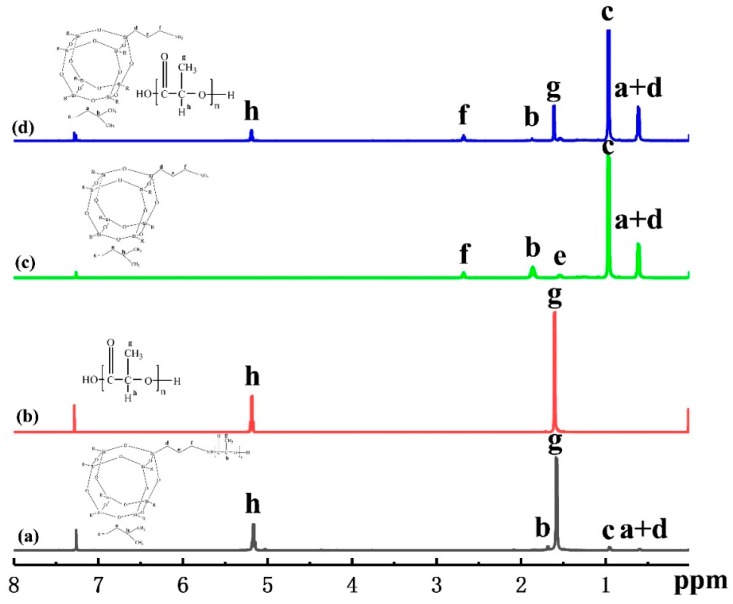
^1^H-NMR spectra of (**a**) PLLA-AMPOSS_0.50_ intermediate, (**b**) neat PLLA, (**c**) AMPOSS, (**d**) PLLA/1wt%AMPOSS blend.

In order to investigate the chemical structure and composition, ^1^H-NMR spectroscopy was performed on the fabricated PLLA-AMPOSS intermediate, neat PLLA, AMPOSS, and simple blend of PLLA and AMPOSS, as shown in Figure 2a–d, respectively. All protons of AMPOSS and PLLA have their own characteristic signals under ^1^H-NMR spectroscopy. AMPOSS (CDCL_3_, ppm) and PLLA resin have two main signals, (SiCH_2_CH(CH_3_)_2_ 0.95) and ((SiCH_2_CH(CH_3_)_2_, SiCH_2_CH_2_CH_2_NH_2_ 0.61), and (OC(CH)CH_3_O 1.58) and (OC(CH)CH_3_O 5.17), respectively. Therefore, the resonance signals that appeared at 0.95, 0.61, 1.58, and 5.17 ppm, shown in Figure 2d, can be attributed to the peaks c and a + d of AMPOSS nanoparticles and g and h of PLLA resin, respectively. Hence, for the simple blend of PLLA/AMPOSS, the signal can be viewed as a simple sum of its components. However, there is a distinct difference in the signals between the PLLA/AMPOSS blend the PLLA-AMPOSS intermediate, although the content of AMPOSS is close in both. Compared to the results shown in Figure 2d, almost all the main signals of AMPOSS can be found to have shifted and weakened for the PLLA/AMPOSS_0.50_ intermediate, as shown in Figure 2a, indicating that the chemical reaction occurred, and the detected signals can be attributed to the resulting ring-opening polymerization products. It can be further verified that the weak peaks f and e of AMPOSS nanoparticles cannot be detected in Figure 2a at all. According to a suggestion by Liu. et al. [32,47], the occurrence of the signal shift and reduction shown in Figure 2a should be attributed to the effect of ring-opening polymerization between the PLLA resin and the AMPOSS particles. Therefore, it can be concluded that the PLLA-AMPOSS intermediate consists of ring-opening polymerization between the PLLA resin and the AMPOSS particles. 

**Figure 3:**
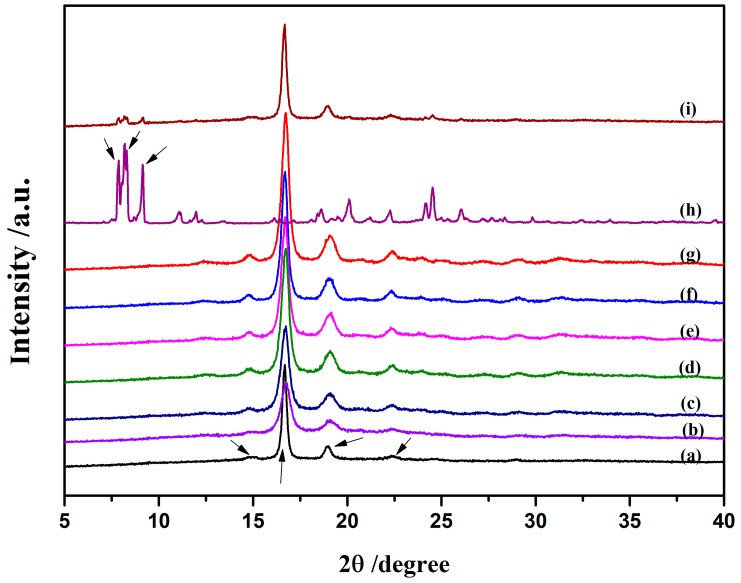
XRD patterns for neat PLLA, PLLA-AMPOSS intermediates, AMPOSS, and PLLA/AMPOSS blends of various concentrations: (**a**) PLLA, (**b**) 0.02 mol%, (**c**) 0.05 mol%, (**d**) 0.10 mol%, (**e**) 0.20 mol%, (**f**) 0.50 mol%, (**g**) 1.00 mol%, (**h**) AMPOSS, and (**i**) PLLA/1wt%AMPOSS blend.

Figure 3 shows the X-ray diffraction patterns of the neat PLLA and PLLA-AMPOSS intermediates with different contents of AMPOSS. As shown in Figure 3a, the strong peaks located at 2θ of 16.8° and 19.0° as well as the weak peaks at 15.0° and 22.5° are typical X-ray diffraction peaks of PLLA, which agrees well with the data reported in [11]. For the AMPOSS nanoparticles, the characteristic peaks were found at many angles, especially the presence of strong signals of 2θ between 6.0° and 9.0°, which can be seen in Figure 3h. It can be seen in Figure 3i that almost all the characteristic peaks of PLLA and AMPOSS can be observed for the simple physical blend of PLLA and AMPOSS (1.0 wt%). This is expected because there is no chemical reaction between the PLLA resin and the AMPOSS nanoparticles. However, it can be also observed from Figure 3b–g that almost all the characteristic diffraction signals of AMPOSS (especially 2θ between 6.0° and 9.0°) were not detected in the PLLA-AMPOSS intermediate at all, no matter how many AMPOSS nanoparticles were incorporated. For PLLA-AMPOSS_0.02_–PLLA-AMPOSS_0.20_ intermediates, the undetectable characteristic peaks are easy to understand and may be attributed to the low content. However, for PLLA-AMPOSS_1.0_, the content of AMPOSS actually exceeded that in the PLLA/AMPOSS blend (1.0 wt%), and the lack of characteristic peaks suggests that the AMPOSS molecule reacted with the PLLA resin. The effect of ring-opening polymerization is thus confirmed. It is also found that the intensity of PLLA peaks increased with increased AMPOSS content, which indicates that the crystallinity of PLLA in the crystallization process increased with increased AMPOSS nanofillers, although the crystalline structure of PLLA was not affected more by the fillers. 

It can be concluded from the NMR and XRD results that synthesizing PLLA-AMPOSS intermediates is feasible and that the reaction of ring-opening polymerization between PLLA and AMPOSS was successful. However, the effect of the PLLA-AMPOSS intermediates on the thermal behavior, crystallization kinetics, and mechanical properties of the fabricated PLLA/PLLA-AMPOSS nanocomposites after the PLLA-AMPOSS intermediates are added into the PLLA matrix by physical blending remains to be explained. This will be clarified as follows.

### 3.2. Structure and Performance of PLLA/PLLA-AMPOSS Composites

#### 3.2.1. Thermal Properties of PLLA and PLLA/PLLA-AMPOSS Nanocomposites

**Figure 4:**
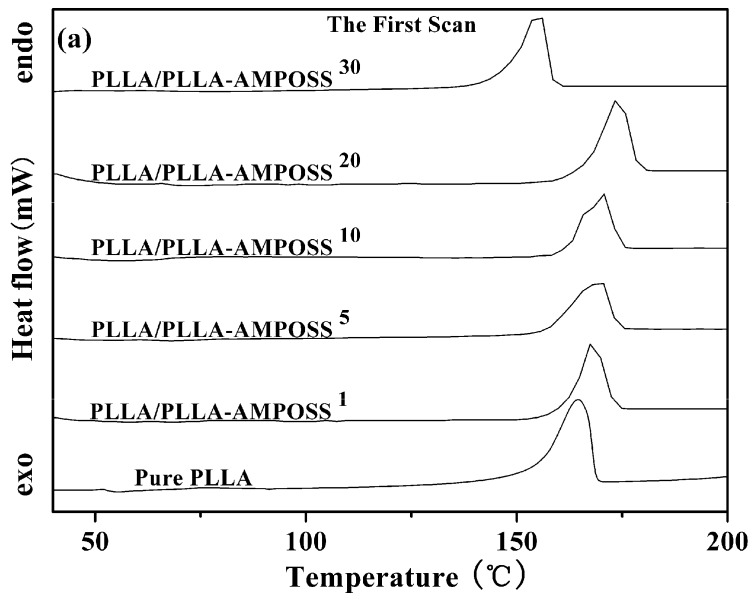

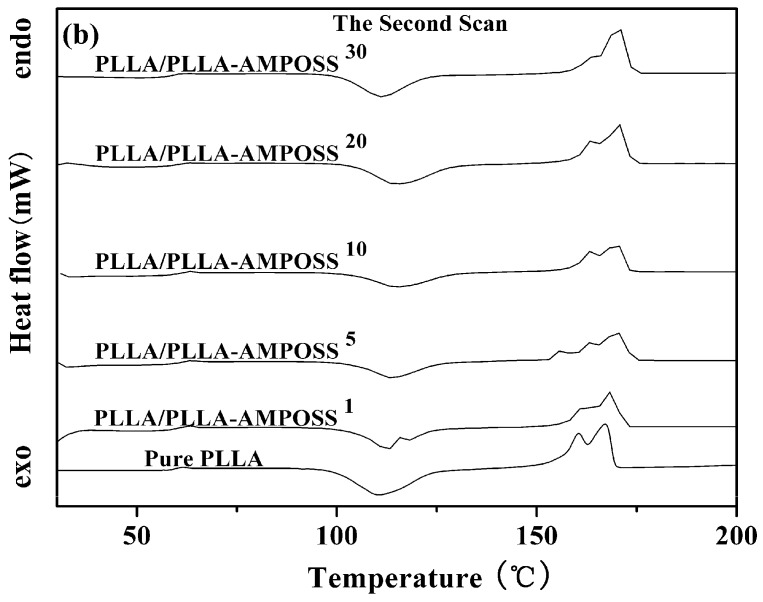
DSC curves of neat PLLA homopolymer and PLLA/PLLA-AMPOSS nanocomposites. (**a**) the first scan, (**b**) the second scan).

The thermal properties of neat PLLA and PLLA/PLLA-AMPOSS nanocomposites were first collected by DSC, as shown in Figure 4. Some information can be obtained from Figure 4, including the glass transition temperature (*T*_g_), the melt peak temperature (*T*_m_), the cold crystallization temperature (*T*_cc_), and the enthalpy of the cold crystallization process (Δ*H*_cc_), which are summarized in Table 1. It can be found that no cold crystallization peaks are detected, whereas melting peaks existed for all tested specimens in the first scanning, as shown in Figure 4a. This is attributed to the crystallization being completed during the dissolution and precipitation of the neat PLLA resin and the PLLA/PLLA-AMPOSS nanocomposites. Compared to the results in the first scanning, the plots of heat flow vs. temperature show different peaks in the second scanning, as shown in Figure 4b. First, a fluctuation of the PLLA/PLLA-AMPOSS nanocomposites at a temperature of 60 °C can be easily observed, and it is known to be the *T*_g_. Second, the cold crystallization peaks at a temperature of approximately 110 °C can be also found for both the neat PLLA resin and the PLLA/PLLA-AMPOSS nanocomposites. Finally, two melting peaks can be clearly observed, and are generally believed to be caused by the two phases of the PLLA crystals [24]. 

Table 1 clearly shows the shift of peak temperature in the DSC thermogram caused by the addition of AMPOSS. Initially, *T*_m_ increased with the increased PLLA/AMPOSS content ranging from 1% to 20%. However, once the content exceeded 20%, the *T*_m_ value decreased instead. Therefore, it can be concluded that the content of PLLA/AMPOSS intermediates had an impact on the *T*_m_ values of the PLLA matrix. There are two competitive effects to explain this phenomenon. On the one hand, the rigid structure of cage AMPOSS and the hydrogen bond of PLLA/AMPOSS probably limit the movement of PLLA macromolecular chains, leading to higher *T*_m_ values compared to the neat PLLA matrix. On the other hand, the LLA chains in the PLLA/AMPOSS intermediate have an effect of plasticization, which results in improvement of the chain mobility, hence *T*_m_ decreased when the content of PLLA/AMPOSS intermediate exceeded 20%. Similar to the variation of *T*_m_, *T*_g_ of the PLLA matrix also increased with increased PLLA/AMPOSS intermediate content, and this is also attributed to the constraint of cage AMPOSS, which probably hinders the motion of molecular segments. As mentioned before, improvement of the *T*_g_ value gives a hint that the hydrogen bond taking place in the carbonyl group with the amino of PLLA and AMPOSS leads to the cross-linked physical structure. The more activation energy arises, the more the *T*_g_ value increases. 

However, it must be noted that the variation of *T*_g_ and *T*_m_ still does not seem to be remarkable according to the above results, which are mainly obtained by the melting process. This behavior may be caused by many others factors affecting the melting process of the nanocomposites. In fact, the main effect of PLLA-AMPOSS is the nucleation effect of the PLLA/PLLA-AMPOSS nanocomposites. A direct comparison of the formation process of crystals is convenient and effective. It is obvious that the enthalpy of the melting process (Δ*H*_m_) of PLLA/PLLA-AMPOSS nanocomposites is higher than that of the neat PLLA resin, which is attributed to the increased crystallinity of PLLA caused by the nucleating effect of AMPOSS nanoparticles. A further investigation of the effect of PLLA-AMPOSS intermediate on crystallization behavior is clarified as follows.

#### 3.2.2. Morphology and Structure of Crystallization 

**Figure 5:**
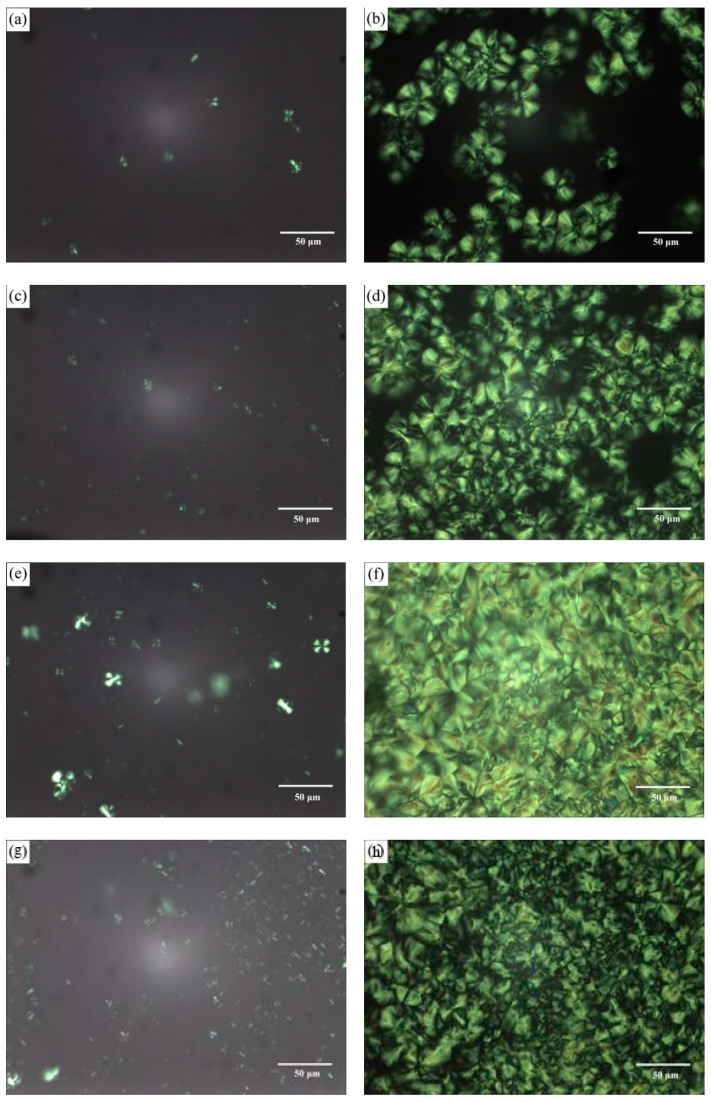
Polarized optical microscopy of crystallization process of PLLA/PLLA-AMPOSS with different PLLA-AMPOSS content and crystallizing time at 125 °C: (**a**) PLLA/PLLA-AMPOSS^1^, 40 s; (**b**) PLLA/PLLA-AMPOSS^1^, 300 s; (**c**) PLLA/PLLA-AMPOSS^10^, 40 s; (**d**) PLLA/PLLA-AMPOSS^10^, 300 s; (**e**) PLLA/PLLA-AMPOSS^20^, 40 s; (**f**) PLLA/PLLA-AMPOSS^20^, 300 s; (**g**) PLLA/PLLA-AMPOSS^30^, 40 s; and (**h**) PLLA/PLLA-AMPOSS^30^ 300 s.

The nucleating effect of PLLA-AMPOSS intermediates on the crystallization of PLLA matrix was also investigated using POM measurements, and the results are shown in Figure 5. The four selected samples, PLLA/PLLA-AMPOSS^1^, PLLA/PLLA-AMPOSS^10^, PLLA/PLLA-AMPOSS^20^, and PLLA/PLLA-AMPOSS^30^ nanocomposite, were melted at 200 °C and cooled down to 125 °C rapidly, and then kept for 40 s and 3 min, respectively. It can be seen from Figure 5 that there are obvious differences in the morphological evolution of the crystallization of the four samples. Not only the number of spherulites but also the rate of nucleating obviously increased with the increased AMPOSS content in a range of 1 wt% and 20 wt%, indicating that the PLLA-AMPOSS intermediate is an effective nucleating agent and possesses excellent crystal-refining effects. 

It must be noted that there are two main factors that influence the nucleating rate of PLLA/PLLA-AMPOSS nanocomposites. AMPOSS nanoparticles can act as sites of heterogeneous nucleation along with the decreased free energy when they are added to the PLLA matrix. Furthermore, the added PLLA-AMPOSS intermediates can induce PLLA chain folding and accelerate the crystallization rate of PLLA/PLLA-AMPOSS nanocomposites. However, a comparison between the results of PLLA/PLLA-AMPOSS^20^ and PLLA/PLLA-AMPOSS^30^ shows that the number and size of their spherulites are almost the same, indicating that the crystallization rate cannot increase further if the content of PLLA-AMPOSS exceeds a certain value. 

#### 3.2.3. Isothermal Crystallization Kinetics 

**Figure 6:**
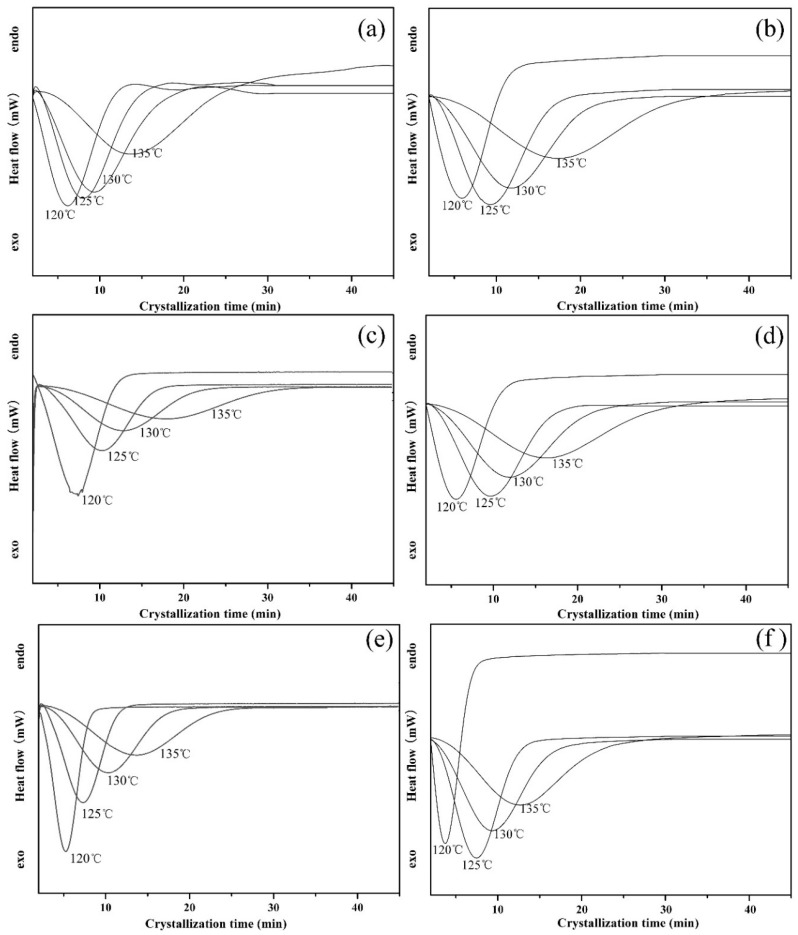
Effects of PLLA-AMPOSS content and crystallization temperature on the isothermal crystallization kinetics of (**a**) pure PLLA, (**b**) PLLA/PLLA-AMPOSS^1.0^, (**c**) PLLA/PLLA-AMPOSS^5.0^, (**d**) PLLA/PLLA-AMPOSS^10^, (**e**) PLLA/PLLA-AMPOSS^20^, and (**f**) PLLA/PLLA-AMPOSS^30^ nanocomposites.

To further clarify the accelerated effect of PLLA-AMPOSS intermediate, the DSC spectrum of isothermal crystallization behavior of neat PLLA and PLLA/PLLA-AMPOSS nanocomposite is shown in Figure 6. In Figure 6, the heat flow is expressed as a function of time. The samples were melted at various temperatures ranging from 120 to 135 °C, which is an isothermal crystallization process. It can be seen in Figure 6 that a high crystallization temperature (*T*_c_) leads to a slow crystallization process, and vice versa, no matter what the content of PLLA-AMPOSS intermediate is. A delay of crystallization induced by increased crystallization temperature is expected. It is partially because the rate of crystallization strongly depends on the nucleation process, including homogeneous nucleation, heterogeneous nucleation, self-nucleation, and the geometry of the growing crystal. According to classic thermomechanical theory, the difference in free energy between the liquid and solid phase, the main driving force of crystallization, is relatively small at high crystallization temperatures. Therefore, a high crystallization temperature means difficulty forming the nucleation and a slow crystallization rate. In addition, it can be also seen in Figure 6 that the existence of PLLA-AMPOSS intermediates can promote the crystallization rate, and the crystallization rate varies depending on the content of PLLA-AMPOSS intermediates. It can be concluded that the more PLLA-AMPOSS intermediates are incorporated, the faster the crystallization will be. However, it can also be observed from a comparison between Figure 6e,f that the crystallization rate cannot increase further if the content of PLLA-AMPOSS exceeds 20%. These results further verify the previous discussion, i.e., the nucleation effects of PLLA-AMPOSS intermediates are very effective when the content does not exceed 20%, and the efficiency decreases gently with excessive filling. It can be concluded from the promoting effects of PLLA-AMPOSS intermediates on the crystallization kinetics that the proposed fabrication method of combining ring-opening polymerization and solution blending is effective to disperse the AMPOSS nanoparticles uniformly in the PLLA matrix. 

The isothermal crystallization kinetics of neat PLLA and PLLA/PLLA-AMPOSS nanocomposite were further analyzed, and the corresponding results are shown in Table 2. It is obvious that the crystallization half-time, *t*_1/2_, in Table 2 is dependent on the weight content of the PLLA-AMPOSS intermediates, i.e., the more PLLA-AMPOSS intermediate added, the smaller the *t*_1/2_ of PLLA/PLLA-AMPOSS nanocomposites. Therefore, it can be concluded that the existence of PLLA-AMPOSS intermediates can accelerate the crystallization rate of PLLA/PLLA-AMPOSS nanocomposites. This means that PLLA-AMPOSS intermediate is an effective nucleation agent of the PLLA matrix. Furthermore, according to the Avrami theory, the crystallization process can be expressed as follows:

(1) $$1-X_{c}=e^{-kt^{n}},$$

where *n* is the Avrami constant, which depends on the style of nucleation and growth mechanism of the crystals, and K is a parameter of crystallization rate. *X*c is the relative degree of crystallization, hence 1 − *X*_c_ represents the ratio of noncrystallization. The *n* and *k* values can be directly obtained from the fitting of the double logarithmic curves and expressed as a slope of 30–70% relative crystallization and the intercept, respectively. These results are listed in Table 2. It can be observed from Table 2 that the exponent *n* of PLLA/PLLA-AMPOSS nanocomposite varies from 2.95 to 3.77. The variation of *n* can be attributed to the secondary crystallization, the density of spherulites, the nucleation process and growth mechanism, and the restraint of intermediate materials. These results combined with the POM measurement results suggest that the crystallized type of pure PLLA resin is homogeneous nucleation and disk-type two-dimensional growth. However, adding the PLLA-AMPOSS intermediate changes the nucleation mode to heterogeneous nucleation and the growth style is three-dimensional spherical. Therefore, the addition of PLLA/PLLA-AMPOSS nanocomposites changed the nucleation mode and growth mechanism of the PLLA. 

**Figure 7:**
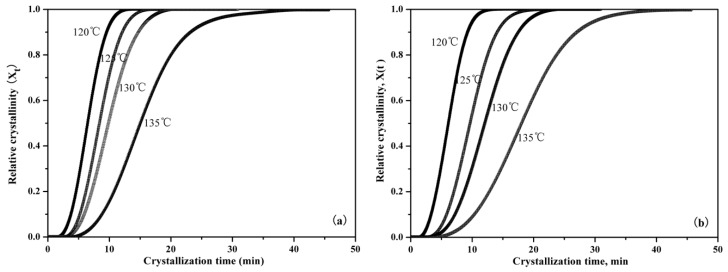

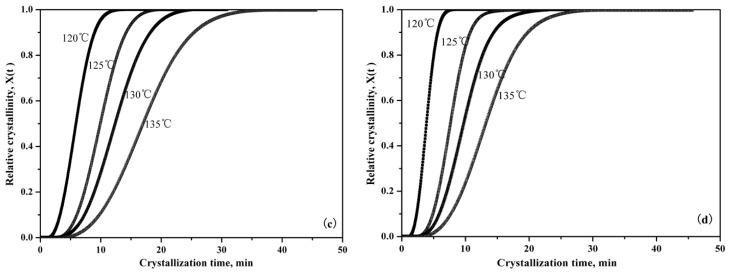
Effects of PLLA-AMPOSS content and crystallization temperature on the relative crystallinity of (**a**) pure PLLA, (**b**) PLLA/PLLA-AMPOSS^1.0^, (**c**) PLLA/PLLA-AMPOSS^10^, and (**d**) PLLA/PLLA-AMPOSS^30^ nanocomposites.

Figure 7 is the representative integral results, showing that relative crystallinity can be expressed as a function of crystallization time during the isothermal crystallization of pure PLLA and PLLA/PLLA-AMPOSS composites. It can be seen from Figure 7 that the curves move to the left with decreased crystallization temperature, which indicates that less time is required to attain the same relative degree of crystallinity. The slope of the isothermal crystallization temperature curve, which is related to the crystallization rate, was also found to be increased with the decreased crystallization temperature. In addition, with a higher content of PLLA-AMPOSS intermediates, an earlier change of crystallization rate takes place, which is in agreement with the previous analysis in Table 2. The efficiency of the PLLA-AMPOSS intermediate was hence further proved.

**Figure 8:**
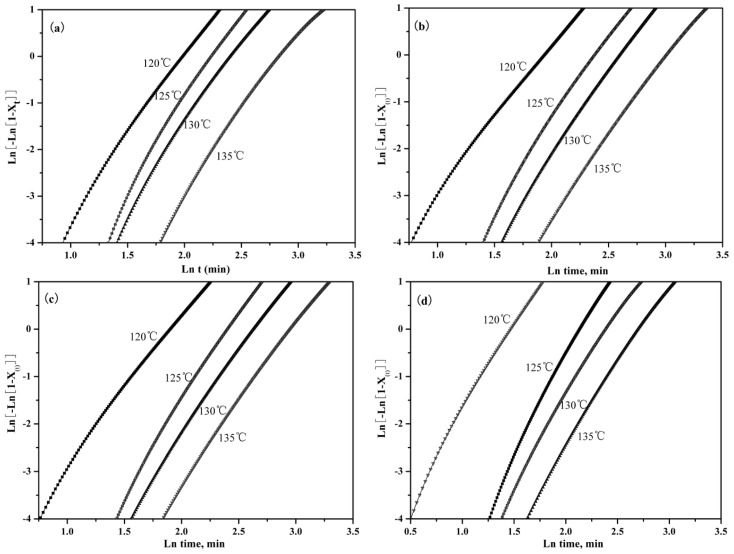
Effects of PLLA-AMPOSS content and crystallization temperature on the Avrami plots of (**a**) pure PLLA, (**b**) PLLA/PLLA-AMPOSS^1.0^, (**c**) PLLA/PLLA-AMPOSS^10^, and (**d**) PLLA/PLLA-AMPOSS^30^ nanocomposites.

The effects of PLLA-AMPOSS content on the plot of $\ln\left\{-\ln\left[1-\left(I_{t}-I_{0}\right)/\left(I_{\infty }-I_{0}\right)\right]\right\}$ versus $lnt_{c}$ can be seen in Figure 8. It can be seen from the good linear relationship shown in the curves of Figure 8 that the variation of the overall crystallization rate depends on the change of crystal growth rate and nucleation rate. The line also moved left, indicating that the crystallization time decreased with the decreased crystallization temperature. It can be also seen that almost all fitted lines in Figure 8 are parallel to each other, which gives a hint that the isothermal crystallographic process at different temperatures was almost the same.

### 3.3. Morphological Characterization of Nanocomposites 

**Figure 9:**
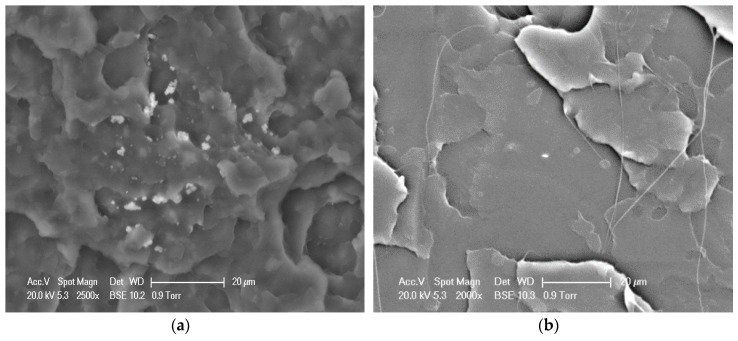
SEM images of fracture surfaces of (**a**) PLLA/0.1wt%AMPOSS blend and (**b**) PLLA/PLLA-AMPOSS^30^ nanocomposite (scale bar is 20 µm).

It is known that the dispersion of POSS in the polymer matrix as a dominant role could affect the physical properties of biodegradable polymers. Therefore, the fracture surface morphology of the physical blend of PLLA/AMPOSS and the fabricated PLLA/PLLA-AMPOSS^30^ nanocomposites samples was determined by SEM, as shown in Figure 9. It can be observed in Figure 9a that several white particles dispersed randomly among the black matrix, which can be realized as AMPOSS nanoparticles, indicating that inhomogeneous dispersion occurred in the simple physical blend of PLLA and AMPOSS, although the content of AMPOSS was as low as only 0.1 wt%. However, compared to Figure 9a, a uniform dispersion of PLLA-AMPOSS can be found in the PLLA matrix and there is good compatibility between copolymerized PLLA-AMPOSS and the PLLA matrix, as shown in Figure 9b. 

### 3.4. Thermal Stability of Nanocomposites

**Figure 10:**
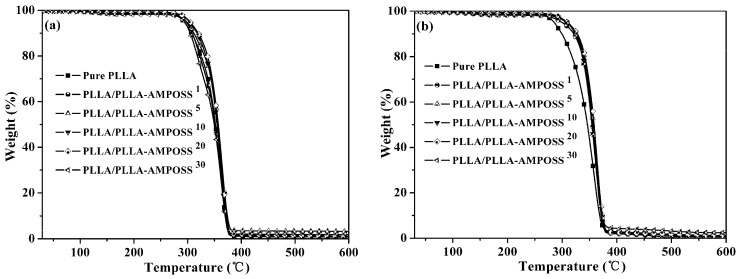
TGA curves of neat PLLA homopolymer and PLLA/PLLA-AMPOSS nanocomposites under (**a**) nitrogen gas and (**b**) air gas conditions.

The thermal stability results of the samples tested under nitrogen gas flow and oxygen conditions are plotted in Figure 10a,b, respectively. The thermo-oxidative decomposition temperatures for 5% and 50% weight loss (*T*_0.05_ and *T*_0.50_) are listed in Table 3. It is obvious that the values of *T*_0.05_ and *T*_0.50_ for neat PLLA are approximately 295 °C and 392 °C in nitrogen atmosphere, and 286 °C and 346 °C in air atmosphere, respectively, and the value of *T*_0.05_ for the PLLA/PLLA-AMPOSS nanocomposites is generally higher than that of pure PLLA. It can be seen in Table 3 that the *T*_0.05_ and *T*_0.50_ values for PLLA/PLLA-AMPOSS nanocomposites increased 16–25 °C and 8–11 °C, respectively, over the neat PLLA resin. Furthermore, the *T*_0.05_ and *T*_0.50_ values of PLLA/PLLA-AMPOSS nanocomposites in air atmosphere are slightly higher than those in the nitrogen atmosphere. The residual amount of weight loss is also seen to gradually increase with the increased PLLA-AMPOSS mass fraction. Therefore, a conclusion can be drawn that there was a tremendous improvement in thermal stability when the content of PLLA-AMPOSS intermediates was increased in a range of 0–20%. In addition, the PLLA-AMPOSS intermediate materials had better improvement of thermal and thermo-oxidative stability in air than nitrogen atmosphere for the PLLA matrix. These results can be attributed to three possible reasons. First, the cage structure of AMPOSS possesses higher thermal stability. Second, a superficial silicified film on the PLLA layer produced by AMPOSS can act as a strong barrier prohibiting gas diffusion caused by small molecules of the material in the thermal degradation process, leading to a delay in degradation. Finally, the end carboxy groups of PLLA resin are converted into hydrogen groups after undergoing a reaction with aminos of the AMPOSS, resulting from the promoting effect of the interaction between the PLLA matrix and PLLA-AMPOSS on the formation of hydrogen groups. However, the thermal stability of the PLLA/PLLA-AMPOSS nanocomposites was also somewhat deteriorated when the additional content of PLLA-AMPOSS intermediates exceeded 20%. This means that, compared with the neat PLLA resin, the lower molecular weight of PLLA-AMPOSS plays a dominant role in decreasing thermo-oxidative stability when the content of PLLA-AMPOSS intermediates is 30 wt%.

It can be seen from the above analysis that the thermal and thermo-oxidative stability of the PLLA/PLLA-AMPOSS nanocomposite was strongly enhanced with the addition of PLLA-AMPOSS intermediate. The effect of the existence of PLL-AMPOSS intermediate was verified again. 

### 3.5. Mechanical Properties of the Nanocomposites

**Figure 11:**
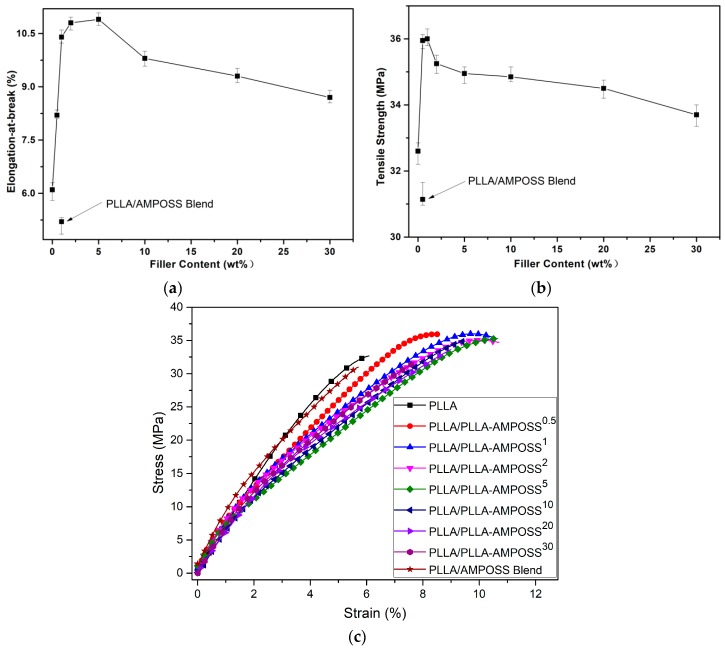
Effects of filler content on (**a**) tensile strength, (**b**) elongation at break, and (**c**) stress–strain plot of PLLA/PLLA-AMPOSS nanocomposites and PLLA/0.1wt% AMPOSS blend.

Using the combined method of ring-opening polymerization and solution blending, nanocomposites of PLLA/PLLA-AMPOSS were prepared and the effect of different contents of PLLA/AMPOSS intermediates on the tensile strength, elongation at break, and stress–strain behavior of the nanocomposites were further investigated, as shown in Figure 11. From Figure 11, it can be seen that both the tensile strength and elongation at break of the composites were improved by incorporating PLLA/AMPOSS intermediates. It is accepted that mechanical properties of semi-crystalline polymer are deeply influenced by the crystallization behaviors. Hence, the improvement in tensile strength and elongation at break can be attributed to the accelerated crystallization process and the reduction in crystal size. However, it must be noted that the effects of different contents of PLLA/AMPOSS intermediates on the mechanical properties and crystallization kinetics are not fully equivalent. When the contents of PLLA/AMPOSS intermediates add up to 1.0%, the tensile strength reaches the peak value, and at 5.0% the maximum elongation at break is shown. The improvement in tensile strength can be attributed to the PLLA/AMPOSS intermediates acting as crosslinking spots in the matrix of the PLLA resin, which limits the molecular deformation during the tensile test. Hence, the PLLA/PLLA-AMPOSS nanocomposites can tolerate some of the tensile force, and the crack will change direction when it comes across the nanoparticles. As a result, the crack can be lengthened and the tensile strength can be improved. Furthermore, the improved elongation at break contributes to the evenly dispersed PLLA/AMPOSS intermediates with PLLA, forming a super interface that has good compatibility. Therefore, both the strength and toughness of the PLLA/PLLA-AMPOSS nanocomposites can be improved simultaneously. A comparison of mechanical properties between neat PLLA and PLLA/AMPOSS simple blend, as shown in Figure 11, can further prove that the nonuniform dispersion of AMPOSS nanoparticles deteriorated the mechanical properties of the PLLA matrix. 

In addition, it can be observed from the stress–strain behaviors shown in Figure 11c that the absorbance energy, which can be expressed as the area of the zone below the stress–strain curve, improved dramatically with the addition of PLLA/AMPOSS intermediates, indicating that the fillers have a remarkable reinforcement and toughening effect. For example, when the content of PLLA/AMPOSS intermediate was 5%, the absorbance energy was approximately twice as high as that of neat PLLA resin. However, with the increased content of PLLA/AMPOSS intermediates, especially, the content of PLLA-AMPOSS intermediates exceeded 20%, and the area showed a gentle decreasing trend. This means that too many PLLA/AMPOSS intermediates can be accumulated, making more cracks emerge in the interface of PLLA and PLLA-AMPOSS intermediate, leading to decreased tensile strength and elongation at break instead. 

Generally, the conventional strengthening or toughening technologies possibly account for this and lose that [15,24,48,49,50,51]. However, it can be seen in Figure 11 that tensile strength and strain at break of the PLLA/PLLA-AMPOSS nanocomposites can be enhanced simultaneously once the optimal dosage of PLLA/AMPOSS intermediates is selected. It can be concluded that the effects of PLLA/AMPOSS intermediates on the mechanical properties of PLLA/PLLA-AMPOSS nanocomposites are remarkable. In addition, it can be found from a comparison of NMR, XRD, SEM, and tensile test between the proposed method and the conventional physical blending method that ring-opening polymerization can successfully incorporate AMPOSS nanoparticles into the PLLA matrix uniformly and the mechanical properties of the PLLA resin can be effectively improved owing to the existence of PLLA-AMPOSS intermediates. Considering that the AMPOSS content was actually very low in the fabricated PLLA/PLLA-AMPOSS nanocomposites using the combination of in situ polymerization and physical blending in this study, the addition of AMPOSS nanosized particles in moderation can be considered a practical and efficient method. 

## 4. Conclusions

In this study, a combined method of ring-opening polymerization and solution blending was proposed, and PLLA/PLLA-AMPOSS nanocomposites were prepared and fabricated. The effect of AMPOSS nanoparticles on the microstructure and performance of PLLA resin were carefully investigated via ^1^H-NMR, XRD, TGA, DSC, SEM, and tensile test. It can be concluded that incorporating AMPOSS nanoparticles can improve the thermal stability, crystallization rate, and tensile properties of the PLLA matrix. Compared with the conventional physical blending of PLLA and AMPOSS nanoparticles and neat PLLA resin, the proposed method was proven to be an effective way to disperse nanosized AMPOSS particles into the PLLA matrix. A suitable use of AMPOSS nanoparticles was also suggested. 

## Figures and Tables

**Table 1:** Differential scanning calorimetry (DSC) data of neat PLLA and PLLA/PLLA-AMPOSS nanocomposites.

Sample	First Scan	Second Scan					
	*T*_m_ (°C)	*T*_g_ (°C)	*T*_cc_ (°C)	Δ*H*_cc_ (J/g)	*T*_m_(°C)		Δ*H*_m_ (J/g)
					*T*_m1_	*T*_m2_	
Pure PLLA	164.6	59.8	110.5	38.1	160.5	167.2	42.2
PLLA/PLLA-AMPOSS^1^	167.4	60.0	112.7	41.7	160.8	168.3	42.9
PLLA/PLLA-AMPOSS^5^	169.5	61.3	113.7	41.8	163.1	169.8	46.0
PLLA/PLLA-AMPOSS^10^	170.7	60.9	115.1	44.0	163.3	169.8	47.3
PLLA/PLLA-AMPOSS^20^	173.3	60.3	114.9	44.2	163.3	170.1	48.0
PLLA/PLLA-AMPOSS^30^	156.0	59.2	111.3	40.6	160.7	170.3	48.8

**Table 2:** Isothermal crystallization kinetics of PLLA/PLLA-AMPOSS nanocomposites.

Samples	*T*_c_ (°C)	*n*	*k* (°C min^−1^)	*t*_1/2_
Pure PLLA	120	3.19	1.43 × 10^−3^	6.93
	125	3.29	2.94 × 10^−4^	10.6
	130	3.15	2.08 × 10^−4^	13.2
	135	3.05	7.68 × 10^−5^	19.8
PLLA/PLLA-AMPOSS^1^	120	3.01	2.95 × 10^−3^	6.15
	125	3.38	3.18 × 10^−4^	9.74
	130	3.35	1.62 × 10^−4^	12.1
	135	3.15	7.36 × 10^−5^	18.3
PLLA/PLLA-AMPOSS^5^	120	2.98	3.13 × 10^−3^	6.12
	125	3.42	2.80 × 10^−4^	9.82
	130	3.31	1.71 × 10^−4^	12.3
	135	3.15	8.27 × 10^−5^	17.6
PLLA/PLLA-AMPOSS^10^	120	2.95	3.63 × 10^−3^	5.93
	125	3.51	2.16 × 10^−4^	9.99
	130	3.28	1.78 × 10^−4^	12.5
	135	3.16	8.67 × 10^−5^	17.2
PLLA/PLLA-AMPOSS^20^	120	3.17	7.02 × 10^−3^	3.82
	125	3.68	3.75 × 10^−4^	7.58
	130	3.29	3.39 × 10^−4^	9.69
	135	3.21	2.18 × 10^−4^	13.03
PLLA/PLLA-AMPOSS^30^	120	3.38	6.90 × 10^−3^	3.91
	125	3.77	3.13 × 10^−4^	7.72
	130	3.31	3.60 × 10^−4^	9.84
	135	3.19	1.75 × 10^−4^	13.4

**Table 3:** TGA data of neat PLLA and PLLA/PLLA-AMPOSS nanocomposites.

Sample	Nitrogen Gas Condition			Air Gas Condition		
	*T*_0.05_ (°C)	*T*_0.50_ (°C)	Residue (%)	*T*_0.05_ (°C)	*T*_0.50_ (°C)	Residue (%)
Pure PLLA	295	352	1.11	286	346	0.73
PLLA/PLLA-AMPOSS^1^	298	352	0.59	302	355	1.05
PLLA/PLLA-AMPOSS^5^	299	357	0.78	311	357	3.23
PLLA/PLLA-AMPOSS^10^	305	355	0.91	311	355	1.01
PLLA/PLLA-AMPOSS^20^	306	357	1.08	311	358	1.12
PLLA/PLLA-AMPOSS^30^	296	350	2.44	310	354	1.84

## Supplementary Materials

Supplementary File 1
