# Supplementary material for: Improving the Thermal and Mechanical Properties of Poly(l-lactide) by Forming Nanocomposites with an in Situ Ring-Opening Intermediate of Poly(l-lactide) and Polyhedral Oligomeric Silsesquioxane
Source: Nanomaterials (Basel). 2019 May 15;9(5):748. doi: 10.3390/nano9050748 (PMC6566323; doi:10.3390/nano9050748)
Supplement: Supplementary File 1 [file nanomaterials-09-00748-s001.pdf]

We certify that the following article

Improve the thermal and mechanical properties of poly(L-lactide) by forming nanocomposites with an in-situ ring-opening intermediate of poly(L-lactide) and polyhedral oligomeric silsesquioxane

Xiu-Xiu Lei, Hao Lu, Lei Lu, Hai-Qing Xu, Ying-Guo Zhou *, Jun Zou *

has undergone English language editing by MDPI. The text has been checked for correct use of grammar and common technical terms, and edited to a level suitable for reporting research in a scholarly journal.
MDPI uses experienced, native English speaking editors. Full details of the editing service can be found at
► https://www.mdpi.com/authors/english.

Multidisciplinary
Digital Publishing
Institute

Basel, May 2019

Martyn Rittman, Ph.D.
English Editing Manager
englishediting@mdpi.com
https://www.mdpi.com/authors/english
